# Vertebrate OTOP1 is also an alkali-activated channel

**DOI:** 10.1038/s41467-022-35754-9

**Published:** 2023-01-03

**Authors:** Lifeng Tian, Hao Zhang, Shilong Yang, Anna Luo, Peter Muiruri Kamau, Jingmei Hu, Lei Luo, Ren Lai

**Affiliations:** 1 Key Laboratory of Animal Models and Human Disease Mechanisms of the Chinese Academy of Sciences/Key Laboratory of Bioactive Peptides of Yunnan Province/National & Local Joint Engineering Center of Natural Bioactive Peptides, Kunming Institute of Zoology, Chinese Academy of Sciences, 650223 Kunming, Yunnan China; 2 National Resource Center for Non-Human Primates, Kunming Primate Research Center/National Research Facility for Phenotypic & Genetic Analysis of Model Animals (Primate Facility), Kunming Institute of Zoology, Chinese Academy of Sciences, 650107 Kunming, Yunnan China; 3 University of Chinese Academy of Sciences, 100049 Beijing, China; 4 School of Molecular Medicine, Hangzhou Institute for Advanced Study, University of Chinese Academy of Sciences, 310024 Hangzhou, China; 5 The cancer Hospital of the University of Chinese Academy of Sciences, Institute of Basic Medicine and Cancer (IBMC), Chinese Academy of Sciences, 310022 Hangzhou, China; 6 College of Wildlife and Protected Area, Northeast Forestry University, 150040 Harbin, China; 7 Sino-African Joint Research Center, Kunming Institute of Zoology, Chinese Academy of Sciences, 650223 Kunming, Yunnan China

**Keywords:** Permeation and transport, Sensory processing, Ion channels, Electrophysiology

## Abstract

Although alkaline sensation is critical for survival, alkali-activated receptors are yet to be identified in vertebrates. Here, we showed that the OTOP1 channel can be directly activated by extracellular alkali. Notably, OTOP1 biphasically mediated proton influx and efflux with extracellular acid and base stimulation, respectively. Mutations of K221 and R554 at the S5–S6 and S11–S12 linkers significantly reduced alkali affinity without affecting acid activation, suggesting that different domains are responsible for acid- and alkali-activation of OTOP1. The selectivity for H^+^ was significantly higher in OTOP1 activated by alkali than that by acid, further suggesting that the two activations might be independent gating processes. Given that the alkali-activation of OTOP1 and the required key residues were conserved in the six representative vertebrates, we cautiously propose that OTOP1 participates in alkaline sensation in vertebrates. Thus, our study identified OTOP1 as an alkali-activated channel.

## Abstract (web summary)

pH sensation is critical for survival of vertebrates. Here, authors found six vertebrate OTOP1 channels that were highly conserved and directly activated by extracellular alkali. Key mutations of OTOP1 reduced alkali affinity without affecting acid activation.

## Introduction

Nearly all physiological processes in the body depend on appropriate acid–base balance. Specific proton-permeable channels mediate proton transport across the membrane and regulate cellular and extracellular pH as well as membrane potential^1^. Several proton channels have been identified, including the influenza M2 protein, an essential viral component for cell infection^2^, and the H_V_1 channel, which favors proton extrusion from cells^3–5^. A new proton-selective channel (Otopetrin1, OTOP1) was recently identified, which exhibits little structural similarity to M2, H_V_1, or other ion channels^6^. Activated by acid, OTOP1 is an essential component of sour taste perception and is functionally conserved in *Drosophila*^7–11^. While the underlying mechanisms of acidic pH sensation have been extensively studied, little is known about how animals sense external alkaline pH. Wang et al. recently showed that transmembrane channel-like protein 1 (TMC-1) mediates alkaline sensation in *C. elegans*^12^. However, the alkali-activated primary sensation receptors are yet to be identified in vertebrates.

OTOP1 is highly expressed in brown and white adipose tissue, mast cells, adrenal glands, and vestibular and taste cells^6,13^. In mice and zebrafish, mutant alleles of *Otopetrin1* can lead to nonsyndromic otoconia agenesis and consequent balance deficits without affecting normal peripheral anatomy and neural circuitry^14,15^. OTOP1 is also conserved in the timing, formation, size, and shape of developing otoliths, which are important for vestibular mechanosensory transduction of linear motion and gravity^16^. OTOP1 attenuates obesity-induced adipose tissue inflammation and plays an adaptive role in maintaining metabolic homeostasis in obesity^13^. Furthermore, OTOP1 is functionally conserved and an essential component of sour taste perception^7–11^.

In this work, we investigated the potential function of OTOP1 in alkaline sensation. We found that it was biphasically modulated by acid (pH ≤ 6.0) and alkali (pH ≥ 8.0), mediating proton influx and efflux with extracellular acid and base stimulation, respectively. We also proposed that alkali activation is a shared feature of various vertebrate OTOP1 channels and that alkali-induced gating differs from that induced by acid.

## Results

### OTOP1 is biphasically activated by acid and alkali

**Fig. 1:**
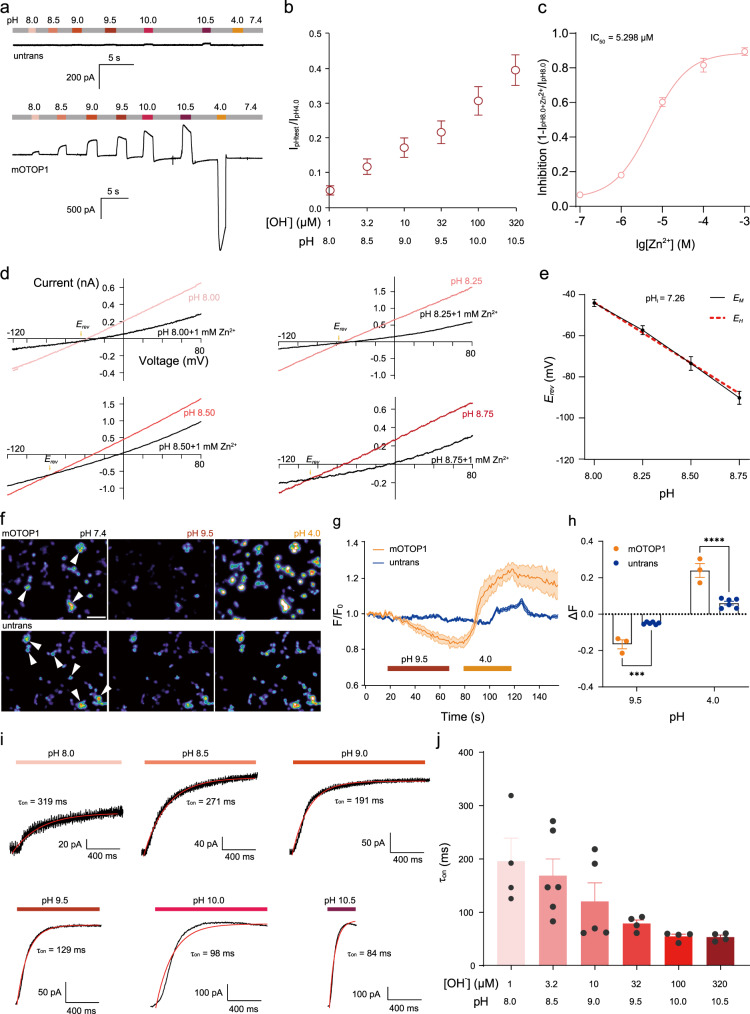
Characterization of mOTOP1 channel alkali activation. **a** Representative current traces induced in mOTOP1-expressing and untransfected HEK293T cells were applied by different alkaline solutions. Untrans, untransfected. **b** Plot of normalized currents (*I*_pHtest_/*I*_pH4.0_) versus pH in OTOP1 channels (mean ± SEM, *n* = 4 cells). Membrane potential (*V*_m_) = 0 mV. **c** Zn^2+^ concentration responses were shown for wild-type mOTOP1 channels. Data points represent mean ± SEM, and the pink line represents the fit of the mean data by a Hill function. *n*  =  3–5 cells. **d** *I*–*V* relationships of mOTOP1 channels were recorded from pH 7.4 extracellular solution to alkaline extracellular solution at different pH values. **e** *E*_rev_ as a function of pH in the experiments of (**d**). The *E*_M_ means were well fitted by linear regression with the slope of −61.86 mV/pH (*R*^2^ = 0.9972). *E*_M_, *E*_Measured_; *E*_H_, *E*_Holding potential_. mean ± SEM. *n*  =  5–9 cells. **f** pH fluorescence images of untransfected and mOTOP1-expressing cells stimulated with pH 4.0 and 9.5. Scale bar, 50 μm. Cells labeled with white arrowheads were used for statistical analysis. **g** Plot of Relative fluorescence intensity (F/F0) versus time in (**f**). mean ± SEM, *n* = 3 of mOTOP1 and *n* = 6 of untransfected. **h** Changes of fluorescence intensity in (**g**). For mOTOP1, *n* = 3; for untransfected, *n* = 6. Two-way ANOVA, Dunnett’s multiple comparisons test, mean ± SEM. Acidic stimuli Δ*F* = *F*_pH4.0_−*F*_0_, *p* = 0.0005; alkaline stimuli Δ*F* = *F*_pH9.5_−*F*_0_, *p* < 0.0001. **i** Time constants of activation (*τ*_on_, red) of mOTOP1 currents (black) in response to different alkaline stimuli (pH 8.0–10.5). Alkaline activation traces of mOTOP1 channel were fitted with a single exponential equation. **j** Summary data for *τ*_on_ of mOTOP1 channel in response to different alkaline extracellular solutions. mean ± SEM, *n* = 4–6 cells. Exact *n* for each data point in (**c**), (**e**), and (**j**) is shown in the “Reproducibility” section in the “Methods” section. ***p < 0.001, ****p < 0.0001. Source data are provided as a Source Data file.

As OTOP1 is considered a proton-selective ion channel, we first characterized the acid-activation properties of OTOP1. Consistent with the previous observations^6^, protons exhibited concentration-dependent induction of the inward macroscopic currents of mouse OTOP1 (mOTOP1) heterologously expressed in human embryonic kidney (HEK293T) cells. As shown in Supplementary Fig. 1a, b, OTOP1 currents increased monotonically as extracellular pH (pH_o_) decreased, indicating that OTOP1 can be functionally expressed and activated by acid. To determine whether OTOP1 responds to other stimuli, we applied alkali to the OTOP1 channels and found that OTOP1 also generated ionic currents. Increasing pH_o_ elicited large outward currents in OTOP1-transfected cells, and the current magnitude increased with pH_o_ (Fig. 1a). The current induced by alkali (pH 10.5) was about 40% of that induced by acid (pH 4.0), with a testing potential of 0 mV (Fig. 1b). Thus, these results suggest that OTOP1 can be directly activated by alkali. As Zn^2+^ has been shown to inhibit OTOP1 channels^6,17^, we wondered whether Zn^2+^ could inhibit alkali-induced currents. As shown in Fig. 1c, Zn^2+^ dose-dependently inhibited pH 8.0-stimulated OTOP1 currents, yielding a half-maximal inhibitory concentration (IC_50_) of 5.30 μM.

We also measured the potential at which the current reverses, i.e., reversal potential (*E*_rev_). For predominantly alkali-induced OTOP1 currents, we used Zn^2+^ to block the OTOP1 currents and focused on the Zn^2+^-sensitive component of the currents. As it is difficult to dissolve Zn^2+^ under alkaline pH, we applied 1 mM Zn^2+^ to the OTOP1 currents evoked by varying alkaline pH. As exhibited in Fig. 1d, e, *E*_rev_ shifted toward more negative voltages with increasing pH_o_, and measured *E*_rev_(*E*_M_) closely followed the Nernst prediction (*E*_H_) for an H^+^-selective ion channel.

### OTOP1 bidirectionally mediates proton influx and efflux

Electrophysiological recordings showed that OTOP1 was activated by both acid and alkali, and protons were transferred inside or outside the cell membrane in response to extracellular acid or alkali stimulation. Furthermore, OTOP1 mediated proton influx and efflux of the cell cytosol, as observed using fluorogenic pH-dependent and membrane-permeant dye pHrodo Red (Fig. 1f). In the OTOP1-transfected cells, but not the mock-transfected cells, increasing extracellular pH from 7.4 to 9.5 resulted in a substantial decrease in pHrodo Red emission. In contrast, decreasing extracellular pH to 4.0 increased pHrodo Red emission (Fig. 1g, h). These results correspond to the electrophysiological recordings (Supplementary Fig. 1a and Fig. 1a, b), showing that the OTOP1 channels bidirectionally mediate proton influx and efflux of the cell cytosol with extracellular acid and alkali stimulation, respectively.

### Alkali gates OTOP1

To further explore whether alkali directly gates OTOP1 channels, we assessed the time course of OTOP1 channels in response to variable alkali concentrations. Tubes with different alkali solutions were placed next to the tube of extracellular solution (pH 7.4), respectively, to ensure the same switching time between different alkaline solutions and the extracellular solution. Activation of OTOP1 in response to pH-variable solutions was measured by fitting the time course of the current upon solution exchange with a single exponential equation. In response to pH 8.0, the time constant for activation of OTOP1 was 195.75 ± 43.29 ms, while the time constants for alkali activation at pH 8.5 and 9.0 were 168.70 ± 31.24 and 120.45 ± 34.66 ms (Fig. 1i), respectively. As the extracellular alkali concentration increased to pH 9.5, 10.0, and 10.5, the time constants for OTOP1 activation were 78.89 ± 6.78, 54.78 ± 3.15, and 53.61 ± 2.79 ms (Fig. 1i), respectively. Theoretically, the time constant for OTOP1 activation at pH 10.5 may be less than pH 10.0 due to greater extracellular alkali concentration. However, we found that the time constant for OTOP1 activation was relatively stable at pH 10.0 and 10.5, which may be the result of another alkali-induced gating process. In fact, we did observe significant current decay with short-term alkali (above pH 9.5) application (Fig. 1i), and long-term alkali (pH 8.0–10.0) application (Supplementary Fig. 2). To explore the extent to which intracellular proton depletion accounts for the decay of the macroscopic currents, current–voltage (*I*–*V*) curves from experiments in which OTOP1 currents were evoked in response to a pH 10.5 stimulus and ramp depolarizations (−120 to +120 mV; 1 V/s) applied for every second showed a shift in *E*_rev_ as a function of time (Supplementary Fig. 3a, b). If the initial current decays due to a change in driving force for H^+^ efflux, the predicted and measured currents should be similar. As shown in Supplementary Fig. 3c, d, the measured magnitudes were less than what can be explained by changes in driving force. Therefore, we tentatively postulated the decay phenomenon is due to intracellular H^+^ depletion and alkali-induced desensitization. In addition, the alkali-induced activation rate of OTOP1 increased with the increase in pH (Fig. 1j). These results suggest that OTOP1 is gated by extracellular alkali.

#### K221 and R554 mutants affect alkali-activation but not acid-activation of OTOP1

**Fig. 2:**
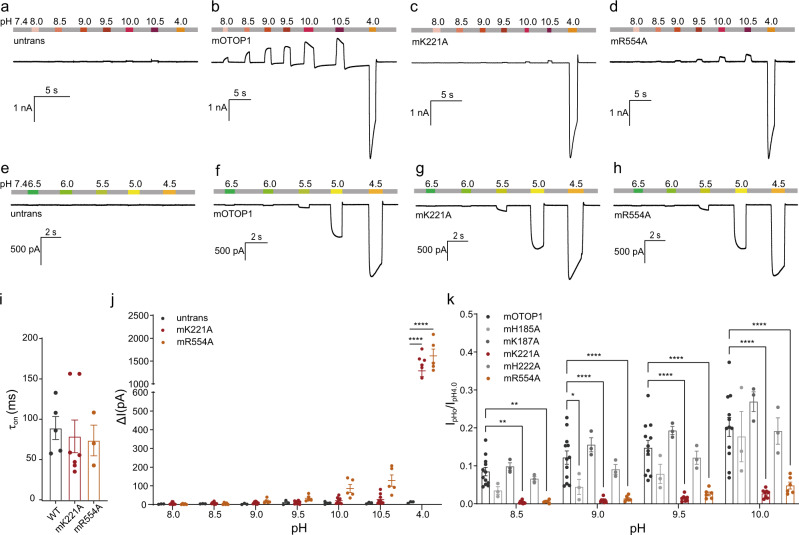
K221 and R554 are key sites for alkali-activation of mOTOP1 but not acid-activation. **a–d** Representative alkali- and acid-induced untransfected (**a**), WT (**b**), mK221A (**c**), and mR554A (**d**) currents with a holding potential of 0 mV. **e–h** Representative traces of currents evoked in untransfected (**e**), WT (**f**), mK221A (**g**), and mR554A (**h**) under different acidic pH (6.5–4.5) OTOP1 extracellular solutions with a holding potential of 0 mV. **i** Summary data for *τ*_on_ of mOTOP1 in response to pH 4.5 extracellular solutions, for WT (*n* = 5), mK221A (*n* = 7), and mR554A (*n* = 3). One-way ANOVA, Dunnett’s multiple comparisons test, mean ± SEM. WT vs. mK221A, *p* = 0.8948; WT vs. mR554A, *p* = 0.8527. **j** Current amplitudes under different pH solutions from experiments as in (**a**), (**c**), and (**d**) (individual data point), for mK221A (*n* = 6), mR554A (*n* = 5), and untransfected (*n* = 3). Two-way ANOVA, Dunnett’s multiple comparisons test, mean ± SEM. Both mutations mK221A and mR554A are significantly different under pH 4.0, compared to untransfected with *p* < 0.0001, respectively. **k** Current magnitudes recorded under variable alkali concentrations were normalized in response to pH 4.0 to control for different cells, for mK187A (*n* = 3), mH222A (*n* = 3), mH185A (*n* = 3), mK221A (*n* = 8), mR554A (*n* = 6), and wild-type (*n* = 13). Two-way ANOVA, Dunnett’s multiple comparisons test, mean ± SEM. *p < 0.05, **p < 0.01, ****p < 0.0001. Exact *p*-values in (**k**): for pH 8.5, **p = 0.0010, **p = 0.0041, for pH 9.0, *p = 0.0492. Source data are provided as a Source Data file.

Due to the binding of alkali to basic amino acids, we performed alanine screening of basic amino acids distributed in the extracellular domain of mOTOP1 to investigate the molecular interactions between alkali and OTOP1. As shown in Fig. 2b, wild-type OTOP1 currents monotonically increased with the increase in pH_o_, while point mutations of K221A (lysine mutated to alanine) in the S5–S6 linker and R554A (arginine mutated to alanine) in the S11–S12 linker largely reduced current amplitude (Fig. 2c, d, and j). We also examined the characteristics of acid-induced activation in the K221A and R554A mutants. Compared with the wild-type OTOP1, neither mutant influenced the magnitude (Fig. 2f–h) and time constant (Fig. 2i) of acid-stimulated inward current activation. When normalized to the inward current generated by pH 4.0, the alkali-induced currents were significantly reduced in the K221A and R554A mutants (Fig. 2k). For instance, at pH 10.0, the alkali-induced current ratios for the K221A and R554A mutants were 2.68% ± 0.39% and 4.80% ± 0.91%, respectively, while the ratio for the wild-type was 20.23% ± 2.51%. Other point mutations, such as H185A and H222A, also reduced the affinity of the alkali–OTOP1 interactions, but not significantly (Fig. 2k). These results suggest that K221 and R554 are candidate residues that affect alkali-activation but not acid-activation of OTOP1, indicating that different domains of OTOP1 may be responsible for acid- and alkali-activation, respectively. In addition, we also co-expressed wild-type mOTOP1 and K221A mutant. As exhibited in Supplementary Fig. 4a, b, the current density of OTOP1 correlated with wild-type OTOP1 expression, which further suggests that K221A, a dominant-negative mutant, is crucial for the alkali-activation of OTOP1.

### OTOP1 permeates multiple cations

**Fig. 3:**
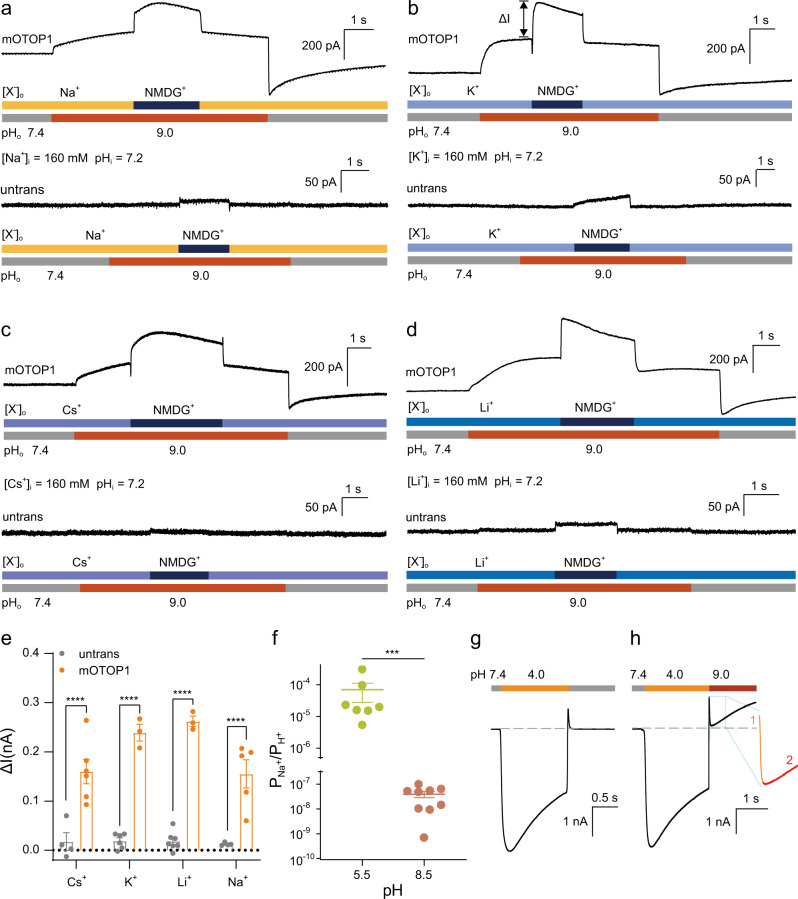
OTOP1 channels permeated multiple cations when activated by alkali. **a–d** NMDG^+^ in extracellular solution was replaced with an equimolar concentration of Na^+^ (**a**), K^+^ (**b**), Cs^+^ (**c**), or Li^+^ (**d**) to measure ionic selectivity of OTOP1. Extracellular solution pH (7.4) was maintained with 160 mM Na^+^, K^+^, Cs^+^, or Li^+^ both inside and outside the cell. The current amplitude increased and was greater than that in the untransfected HEK293T cells when the dosing tube was switched directly from pH 9.0 extracellular solution containing other cations to pH 9.0 extracellular solution containing 160 mM NMDG^+^. [X^+^]_o_, extracellular cation ion concentration; [Na^+^]_i_, intracellular Na^+^ concentration. pH_i_, intracellular pH; pH_o_, extracellular pH. **e** HEK293T cells expressing OTOP1 channels showed significantly higher increases in currents when other cations were permeated compared to untransfected cells. Two-way ANOVA, Dunnett’s multiple comparisons test, mean ± SEM, *n* = 3–7 cells, exact *n* for each data point is shown in the subsection “Reproducibility” in the “Methods” section. For Na^+^, K^+^, Cs^+^, and Li^+^, **** *p* < 0.0001. **f** Ionic permeability ratio of Na^+^ to H^+^ when mOTOP1 channels were activated by acid (pH 5.5, *n* = 7 cells) or base (pH 8.5, *n* = 9 cells). pH 5.5: ${P}_{{{{\rm {Na}}}}^{+}}$/${P}_{{{\rm {H}}}^{+}}$ = (6.973 ± 4.186) × 10^−5^; pH 8.5: ${P}_{{{{\rm {Na}}}}^{+}}$/${P}_{{{\rm {H}}}^{+}}$ = (3.961 ± 1.095) × 10^−8^. Mann–Whitney test, two-tailed, mean ± SEM, ***p = 0.0002. **g** No significant gating activation of OTOP1-activated cells was observed when the extracellular solution pH changed from 4.0 to 7.4. **h** Obvious gating activation in OTOP1-activated cells was observed when extracellular pH changed from 4.0 to 9.0. ***p < 0.001, ****p < 0.0001. Source data are provided as a Source Data file.

We further assessed the ion selectivity of OTOP1 during acid- and alkali-induced activation. To determine whether alkali-activated channels are proton-selective, we evoked OTOP1 currents by increasing pH_o_ from pH 7.4 to 9.0 and measured the effect of exchanging Na^+^, K^+^, Cs^+^, or Li^+^ in the extracellular solution for equimolar concentrations of NMDG^+^. As shown in Fig. 3a, the alkali-activated current increased by nearly 100% after Na^+^ was exchanged with NMDG^+^ in the extracellular solution. Notably, we observed significant current jumps in all four recordings (Fig. 3a–d, top). To exclude cell background interference, we compared OTOP1-transfected and mock-transfected cell currents (Fig. 3a–d, bottom). In all four cases, the changes in current magnitude were significantly higher in the OTOP1-transfected cells than in the mock-transfected cells (Fig. 3e), indicating that alkali-activated OTOP1 is permeable to protons as well as Na^+^, K^+^, Cs^+^, and Li^+^. As seen in Fig. 3a–d, the concentrations of Na^+^, K^+^, Cs^+^, and Li^+^ were much higher than the protons. We, therefore, used the Goldman–Hodgkin–Katz (GHK) equation to calculate the ion selectivity of the acid- and alkali-induced OTOP1 channels. By measuring *E*_rev_ after the replacement of NMDG^+^ by Na^+^ and with high and low concentrations of Na^+^ in the extracellular solution, the selectivity ratio of acid-activated OTOP1 for Na^+^ relative to H^+^ was 6.97 × 10^−5^, while that of alkali-activated OTOP1 was 3.96 × 10^−8^ (Fig. 3f). This is as expected if the regions responsible for acid- and alkali-activation of OTOP1 are independent. We also tested the permeability of Ca^2+^, a cation critical to neuronal activity, on the OTOP1 channel. After substituting NMDG^+^ for various concentrations of Ca^2+^, none of the recordings revealed any substantial current jumps (Supplementary Fig. 5a–d). Statistical analysis showed that the small current variation upon ion exchange was not dependent on Ca^2+^ concentration (Supplementary Fig. 5e). These results suggested that alkali-activated OTOP1 is likely not permeable to Ca^2+^. Furthermore, in the OTOP1-activated cells, the increase in extracellular pH from 4.0 to 9.0 resulted in obvious gating activation (Fig. 3h). In contrast, the increase in extracellular pH from 4.0 to 7.4 did not induce significant gating activation, as expected (Fig. 3g). These results further suggest that alkali-induced activation and acid-induced activation might be independent gating processes.

### Alkaline activation of OTOP1 in physiological conditions

**Fig. 4:**
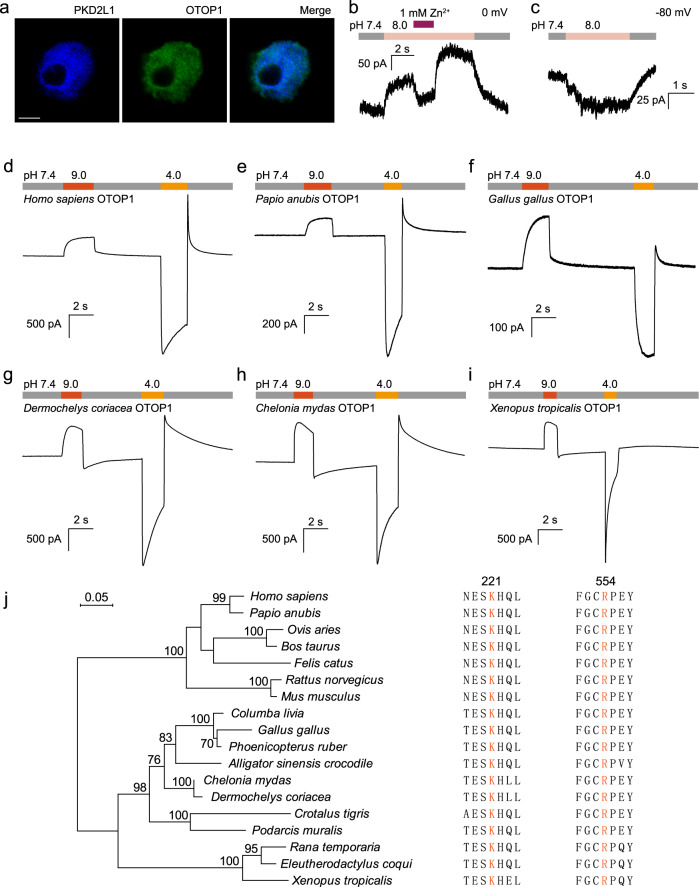
Alkaline activation of OTOP1 channels was relatively conserved in vertebrates. **a** Confocal image showing taste cells from mice with antibodies against OTOP1 (green), and PKD2L1 (blue). Scale bar, 5 μm. **b** Currents in response to pH 8.0 NMDG-based extracellular solution in isolated taste cells. 1 mM Zn^2+^ inhibited outward current generated by pH 8.0 extracellular solution. **c** Inward current was induced in isolated taste cells by pH 8.0 extracellular solution under physiological conditions. **d–i** Representative whole-cell recordings of six vertebrate OTOP1 channels in response to Na^+^-free extracellular solutions with different pH (as indicated), with holding and test voltages both set to 0 mV. **j** Maximum-likelihood phylogenetic tree created from multiple sequence alignment of OTOP1 channel proteins in vertebrates (left). Bootstrap values of branches are marked on nodes, the number of bootstraps was 1000. The scale bar (top, left) indicates 0.05 amino acid substitutions per site. Alignment of amino acid sequences of OTOP1 in vertebrates. Key residues associated with alkali binding are shown in orange (right). Source data are provided as a Source Data file.

Considering alkaline pH induces nociception and gustatory responses in vertebrates^1^, we detected the alkali-induced currents in taste receptor cells (TRCs). OTOP1 was reported to be endogenously expressed in type III TRCs, which were identified by the presence of a unique marker, PKD2L1 protein^7,18^. Our immunofluorescence analysis revealed that the OTOP1 colocalized with PKD2L1, indicating the presence of OTOP1 in TRCs (Fig. 4a). In an artificial setting, we observed outward currents in TRCs upon pH 8.0 application (Fig. 4b), which had similar current characteristics recorded from HEK293T cells heterologously expressing OTOP1 (e.g., Fig. 1a). Similar Zn^2+^ inhibition was also observed (Fig. 4b). To further determine whether OTOP1 contributes to the alkali-induced current in TRCs in a physiological setting, we observed significant inward currents upon alkaline pH application (Fig. 4c), which might underly alkaline sensation in TRCs.

### OTOP1 alkaline activation is conserved in vertebrates

OTOP1 is highly conserved in vertebrates, arthropods, and nematodes^6,19^. Thus, we explored whether alkali-induced activation is conserved in vertebrates. OTOP1 in six representative species (i.e., humans, olive baboons, chickens, leatherback turtles, green sea turtles, and western clawed frogs) was identified in whole-genome sequences, and OTOP1 eukaryotic expression plasmids were constructed. All six representative species exhibited a normal response to extracellular acid. Notably, alkali at pH 9.0 elicited large outward currents in all six species (Fig. 4d–i), suggesting conserved OTOP1 alkaline activation in multiple vertebrates. As basic amino acids K221 and R554 in mOTOP1 are crucial for alkali–OTOP1 interactions, we aligned OTOP1 sequences of the representative vertebrates and found that both K221 and R554 (in mouse OTOP1 number) were highly conserved in amphibians to mammals (Fig. 4j).

## Discussion

Like acid, alkaline pH also induces gustatory responses and nociception^20^. Various channels are known to be activated or modulated by extracellular (TMC-1) or intracellular (TRPV1, TRPA1, N-type calcium channels, HCN2, and CatSper1) alkalization^21–25^. In addition, TRPV1 is the first receptor with a striking ability to be activated by both acidic (extracellular) and basic (intracellular) pH^21,22^. However, the cellular and molecular nature of external alkaline sensations are poorly understood, and no alkali-activated primary receptors have been confirmed in vertebrates^12,20^. Here, we report that extracellular alkali could directly activate OTOP1 in a concentration-dependent manner. OTOP1 was previously considered to be exclusively activated by protons, with selective permeation of protons upon extracellular acid application^6^. Extracellular application of alkali mediated the efflux of multiple cations, such as H^+^, Na^+^, K^+^, Cs^+^, and Li^+^, out of the cytoplasm, which may be related to the maintenance of acid–base balance and excitability of cells, as reported previously^26^. For instance, Otop2l, an OTOP ortholog in sea urchins, promotes cellular acid efflux critical for biomineralization. The ubiquitous occurrence of OTOP families in calcifying systems across the animal kingdom suggests a conserved physiological function, with pH mediated at the mineralization site^26^. Given the deep phylogenetic origin of otopetrins in animals^19^, our results and those of others indicate that the cellular pH sensation and regulation may be inherent features of otopetrins.

We found that mutations of K221 and R554 at the S5–S6 and S11–S12 linkers significantly reduced alkali affinity to the OTOP1 channel. Notably, neither mutation on the extracellular domains altered acid-induced activation, suggesting that different regions of OTOP1 might be responsible for acid- and alkali activation, respectively. Compared with acid-induced OTOP1 currents, alkali-induced currents exhibited higher selectivity for H^+^ relative to Na^+^, further suggesting that acid- and alkali-activation are likely independent gating processes in OTOP1. Based on OTOP1 structural analysis and molecular dynamics, Saotome et al. proposed three potential proton conduction pathways, including the N and C domains and the intrasubunit interface between them^27^. Although the structure of mOTOP1 has not yet been established, AlphaFold-based structural predication of mOTOP1^28^ suggests that K221 and R554 are located at positions near the putative pore within the intrasubunit interface (Supplementary Fig. 6a), which might be involved in alkali activation. Moreover, cryo-EM structure analysis indicates that R540 in zebrafish OTOP1 (equivalent to R554 in mOTOP1) is located at the interface between the N and C domains, producing an electropositive region near the bilayer midplane point of the interface^27^ (Supplementary Fig. 6a, b). These results suggest that the intrasubunit interface may be involved in alkali-induced activation of the OTOP1 channel.

We also measured the currents of naturally occurring mutations (*tilted* and *mergulhador*) in the OTOP1 channel. As shown in Supplementary Fig. 7a, both mutations (*tilted* and *mergulhador*) significantly reduced acid-induced inward current amplitude, as previously reported^6,15^, and slowed the acid-induced rate. We compared all alkali-induced currents for wild-type and mutant OTOP1 by normalizing to pH 4.0 as exhibited in Fig. 2k, whereas the pH 4.0-induced currents for both naturally occurring mutations were significantly reduced and the acid-activation dynamics were changed as compared with wild-type OTOP1. Therefore, we were unable to conclude whether naturally occurring mutations were involved in alkali-induced activation, although the current densities for both mutations (*tilted* and *mergulhador*) were significantly lower than the wild-type OTOP1 channel (Supplementary Fig. 7c). To explore the Zn^2+^ inhibition regions, we performed a voltage-dependent experiment on Zn^2+^ inhibition. As demonstrated in Supplementary Fig. 8, the inhibition efficiency of Zn^2+^ on OTOP1 was not dramatically changed upon different stimulating voltages, indicating that Zn^2+^ might not bind to the pore region of OTOP1^29^. As we proposed above, the intrasubunit interface may be involved in alkali-induced activation of the OTOP1 channel, therefore, Zn^2+^ is likely to bind to extracellular regions other than the intrasubunit interface of OTOP1.

Recently, Teng et al. observed a small outward current in mOTOP1-transfected cells in response to alkaline stimulation (pH 9.0–10.0) with a holding potential at −80 mV. In comparison with our recordings, alkali-induced OTOP1 currents were a little small, which could be explained by the lower holding potential. These results further support the conclusion of alkali-induced OTOP1 activation. Strikingly, mOTOP2 is open at neutral pH and exhibited large outward currents in response to alkaline solutions of pH 9.0 and 10.0, while mOTOP3-transfected cells showed no outward currents in response to any of the alkaline stimuli^30^. Given that both OTOP1 and OTOP2 channels can conduct outward current in response to alkaline stimulation, they are likely to participate in some alkali-related physiological activities.

OTOP1 was the first described member of the otopetrin family, and vertebrate otopetrins share a conserved gene and protein structure^19,31^. Among the six vertebrate species tested, the OTOP1 channels in mammals, birds, reptiles, and amphibians were all alkali-activated, suggesting a conservative pattern of alkali activation in vertebrates. Moreover, key residues (K221 and R554 at S5–S6 and S11–S12 linkers) of OTOP1 that affect alkali affinity were highly conserved in the vertebrates. Consistent with our findings, the S5–S6 and S11–S12 linkers in a refined topological model of the otopetrin family exhibit maximum evolutionary constraints and length conservatism in vertebrates^19^. The high sequence and functional similarity of the OTOP1 channels suggest a conserved role in alkaline pH sensation. This hypothesis is consistent with our observation that extracellular alkali application induced inward currents in a physiological environment (Fig. 4c). The significant inward currents might underly alkali-induced membrane depolarization and associated alkaline sensation. In addition, the application of alkaline solution (pH 11.0–12.5) to mammalian tongues evokes a continuous outburst of impulses in the chorda tympani nerve^20^, and the response of the nerve is essential for chemical sensation in mammals^7,9,32^. Thus, we reasoned that vertebrate OTOP1 might function in alkaline sensation and/or other alkali-related functions. Techniques such as transgenic manipulations or pharmacological interventions may be useful in determining whether OTOP1 contributes to vertebrate alkaline perception. The activation of vertebrate OTOP1 channels by alkaline pH reveals another function of OTOP1.

## Methods

### Animals

All animal experiments were approved by the Institutional Animal Care and Use Committees of the Kunming Institute of Zoology, Chinese Academy of Sciences (approval ID IACUC-RE-2022-08-015). The health status of C57BL/6L mice was monitored daily and only healthy mice were used in experiments.

### Construction of OTOP1 channel plasmids

OTOP1 gene sequences were obtained from the NCBI database for the following species: *Gallus gallus* (Gene ID: 422844), *Xenopus tropicalis* (Gene ID: 100495027), *Chelonia mydas* (Gene ID: 102932521), *Dermochelys coriacea* (Gene ID: 119854908), *Papio anubis* (Gene ID: 101001867), *Mus musculus* (Gene ID: 21906), and *Homo sapiens* (Gene ID: 133060). We optimized OTOP1 gene codons of the above species into codons for human expression systems. The OTOP1 gene sequences were synthesized and cloned into the pcDNA3.1 eukaryotic expression plasmid by Sangon Biotech (Shanghai). After screening positive clones with ampicillin resistance, sequencing was performed to obtain the pcDNA3.1 plasmid containing the correct OTOP1 sequence. All mOTOP1 point mutations were constructed using the Mut Express® II Fast Mutagenesis Kit V2 (Vazyme, C214). Point mutations of mOTOP1 were confirmed by sequencing.

### Cell preparation and transfection

The HEK293T cells were purchased from the Kunming Cell Bank, Kunming Institute of Zoology, Chinese Academy of Sciences. Cells were routinely cultured at 37 °C in 5% CO_2_ and 95% O_2_ in Dulbecco’s modified Eagle medium (DMEM) (Corning, 10-013-CV) containing 10% fetal bovine serum (Corning, 35-076-CV), and 1% penicillin/streptomycin (Gibco). According to the manufacturer’s instructions, the HEK293T cells were transiently transfected with 5 μg of channel plasmid and 0.5 μg of enhanced green fluorescent protein (eGFP) plasmid using Lipofectamine 3000 transfection reagent (Life Technologies, L3000015). After 24 h of transfection, cells with eGFP were selected for patch-clamp recording.

### Taste cells isolation

Preparation of Tyrode’s solution (in mM): 145 NaCl, 5 KCl, 1 MgCl_2_, 2 CaCl_2_, 20 d-glucose, 10 HEPES, pH 7.4 with NaOH. Taste cells were isolated from adult C57BL/6L mice (8–12 weeks). After cervical dislocation and execution of the mice, tongues were removed. A mixture of Tyrode’s solution added with 1 mg/mL Elastase (Worthington Biochemical) and 2.5 mg/mL Dispase II (Sigma Aldrich) was injected between the epithelium and muscle of the isolated tongues from the root to the tip of the tongues, and then incubated in 5% CO_2_ and 95% O_2_ in Tyrode’s solution for 30 min^6^. The tongue epithelium is peeled off and the clipped epithelium with circumvallate papillae is incubated in the enzyme mixture for 50 min at room temperature. Transfer digested circumvallate papillae into the dish with round glass coverslips, and dissect circumvallate papillae gently with a surgical razor in Tyrode’s solution. The isolated taste cells were cultured at room temperature and used for experiments after 3–7 h.

### Electrophysiology

As described previously, macroscopic currents were recorded using a HEKA EPC10 amplifier controlled by PatchMaster software (HEKA). Whole-cell recordings were performed at 0 mV. Patch pipettes were prepared from borosilicate glass (WPI) using a Flaming/Brown Micropipette Puller (SUTTER P-97) and fire-polished to a resistance of 3–5 MΩ by Polisher (NARISHIGETM MF-830). Tube switching data of the gravity-driven system were transmitted to the amplifier in real-time and processed using PatchMaster to record the corresponding current change data of each tube solution in real time. Millisecond solution exchange was achieved with a fast-step perfusion system (RSC-200, Bio-Logic) custom modified to hold microcapillary tubes in a linear array.

For OTOP1 channel recordings, the artificial pipette solutions contained (in mM):160 NMDG-Cl, 2 CaCl_2_, and 10 HEPES (pH adjusted to 7.2 with HCl). The artificial OTOP1 extracellular solutions with different pH in the perfusion system contained (in mM): 160 NMDG-Cl, 2 CaCl_2_, and 10 buffers based on pH (Homo PIPES for pH 4.0–6.5, HEPES for pH 7.4, Tris for pH 8.0–10.5). The artificial setting was used for OTOP1 channel recordings unless otherwise stated. OTOP1 extracellular solutions with different pH were perfused to membrane patch by a fast-step perfusion system.

For the taste receptor cells (TRCs) recordings, the physiological setting was as follows: pipette solution contained (in mM) 140 KCl, 0.5 EGTA, 10 HEPES, 2 Mg-ATP (pH adjusted to 7.3 with KOH), and extracellular solution contained (in mM) 140 NaCl, 3 KCl, 2 CaCl_2_, 2 MgCl_2_, and 10 buffer based on pH (HEPES for pH 7.3, Tris for pH 8.0).

In the ionic selectivity experiment in Fig. 3a–d, 160 mM NMDG-Cl was replaced by equimolar concentrations of LiCl, CsCl, KCl, or NaCl, with 200 µM amiloride added to block endogenous acid-sensing ion channels^6^. Prior to the alkaline extracellular solution (pH 9.0) application, the intracellular and extracellular solutions (pH 7.4) contained 160 mM Li^+^, Cs^+^, K^+^, or Na^+^, respectively. Upon application at pH 9.0, the extracellular solutions were switched from monovalent cations (i.e., 160 mM Li^+^, Cs^+^, K^+^, or Na^+^, respectively) to 160 mM NMDG^+^, yielding enlarged outward currents. In the ion permeability experiment of Ca^2+^ in Supplementary Fig. 5, Calcium ions were chelated intracellularly with 2 mM EGTA, and NMDG^+^ in the extracellular solution was replaced with gradient concentrations of Ca^2+^ under the condition of maintaining the same osmotic pressure.

In Fig. 1e, *E*_rev_ of the alkali-activated current of the mOTOP1 channel in the Zn^2+^-sensitive fraction, is obtained by subtracting the alkali-activated *I*–*V* curve with the addition of Zn^2+^ from the alkali-activated *I*–*V* curve. *V*_m_ was held at −120 mV and ramped to +80 mV. pH 8.00, holding potential was −43.75 mV; pH 8.25, holding potential was −58.54 mV; pH 8.50, holding potential was –73.32 mV; pH 8.75, holding potential was −88.10 mV.

In the *E*_rev_ recordings, posteriori corrections were made to minimize the effects of liquid junction potentials. Liquid junction potentials in Figs. 1d, 3f and Supplementary Fig. 3 were measured between the pipette solution and the bath solution as previously reported^33^ and were corrected posthoc. In order to minimize junction potentials between bath solutions and “sewer pipe”, a 3 M KCl salt bridge, with a well-chlorided silver wire inside, was used as the bath ground. Junction potentials of <2 mV were not corrected.

### Permeability ratio

The ratio of Na^+^ to H^+^ ion permeability when the mOTOP1 channel was activated by acid: After the cells were clamped, the membrane potential was held at *E*_H_ (−78 mV), then ramped from −120 to +80 mV (1 V/s). *E*_rev_ was measured from the Zn^2+^-sensitive part of the current^6^. Zn^2+^ was added to the OTOP1 perfusion extracellular solution as control at a concentration of 10 mM. The pipette solutions contained (in mM): 130 TMA–methane sulfonate, 5 TEA–Cl, 2 Mg–ATP, 5 EGTA, 2.4 CaCl_2_, and 80 MES (pH adjusted to 6.0 with TMA–OH). The OTOP1 extracellular solutions in the perfusion system contained (in mM): 130 Na–methane sulfonate or 130 NMDG–methane sulfonate, 2 CaCl_2_, and 100 MES (pH adjusted to 5.5 with NMDG–OH). 200 µM amiloride was added to the extracellular solutions to block endogenous acid-sensing ion channels.

The ratio of Na^+^ to H^+^ ion permeability when the mOTOP1 channel was activated by alkaline: After the cells were clamped, the membrane potential was held at *E*_H_ (+29 mV), then ramped from −120 to +80 mV (1 V/s). *E*_rev_ was measured from the Zn^2+^-sensitive part of the current. Zn^2+^ was added to the OTOP1 perfusion extracellular solution as a control at a concentration of 1 mM. The pipette solutions contained (in mM): 160 NMDG-Cl, 2 CaCl_2_, and 10 HEPES (pH adjusted to 7.2–7.4 with HCl). The OTOP1 extracellular solutions in the perfusion system contained (in mM): 130 Na–methane sulfonate or 130 NMDG–methane sulfonate, 2 CaCl_2_, and 100 Tris (pH adjusted to 8.5 with HCl).

According to the Goldman–Hodgkin–Katz (GHK) equation^34^, we used the following formulas (1) and (2) to calculate the ion permeability ratio of Na^+^ to H^+^:

1 $$\triangle V={{E}_{{{\rm {rev}}}}}_{0{{\rm {mM}}}{{{\rm {Na}}}}^{+}}-{{E}_{{{\rm {rev}}}}}_{130{{\rm {mM}}}{{{\rm {Na}}}}^{+}}$$

2 $$\frac{{P}_{{\rm {N{a}}}^{+}}}{{P}_{{{\rm {H}}}^{+}}}=\frac{(1-{\rm {exp}}(\frac{zF\Delta V}{RT}))\times {[{H}^{+}]}_{{\rm {o}}}}{{[{\rm {N{a}}}^{+}]}_{{\rm {o}}}\times {\rm {exp}}(zF\Delta V/RT)}$$

where *P* is the permeability of cell membranes to ions, *R* denotes the gas constant, *T* is the temperature, *z* is the ion valency, *F* is Faraday’s constant, *E*_rev_ is the reversal potential.

### pH fluorescence imaging

The pH fluorescence imaging experiments in Fig. 1f were performed using a pHrodo™ Red AM Intracellular pH Indicator Kit (Life Technologies, P35372). The OTOP1 plasmid was transfected into HEK293T cells, incubated overnight in a cell incubator (37 °C in 5% CO_2_ and 95% O_2_), digested, and spread onto cell crawls for at least 1 h. The pHrodo™ Red AM (10 μL) and PowerLoad™ (100 μL) were mixed in 10 mL of OTOP1 channel extracellular solution. The solution (2 mL) was then added to each small dish of cells and incubated at 37 °C for 15–30 min to allow the pH indicator to enter the cells. Cells were stimulated to produce pHrodo™ Red fluorescence using pH 4.0 solution (150 mM NaCl, 10 mM Homo PIPES, and 2 mM CaCl_2_, pH adjusted with HCl) and pH 9.5 solution (150 mM NaCl, 10 mM Tris, and 2 mM CaCl_2_, pH adjusted with HCl)^6^.

After incubation, changes in pH fluorescence of the HEK293T cells were captured using a Hamamatsu C4742 CCD digital camera controlled by MetaFluor Software (Molecular Devices). A 560-nm excitation filter of the LED light source (X-Cite 120LED) was used for the excitation of pHrodo™ Red AM. The pHrodo™ Red AM fluorescence was filtered using a 580-nm emission filter for detection. MetaMorph software (Molecular Devices) was used to process and analyze the fluorescence images. Fluorescence intensity was normalized by the initial fluorescence intensity of each cell. Six sets of fluorescence values were selected during the periods of different stimuli, and after calculating the mean value of fluorescence intensity for each cell, significant differences between mOTOP1 and untransfected cells were statistically analyzed.

### Immunofluorescence

The isolated taste cells were plated onto 10 mm coverslips in Tyrode’s solution for 3–7 h. Then cells were fixed with 4% paraformaldehyde (PFA, Biosharp) in phosphate buffered saline (PBS, Corning) for 15 min and washed three times with PBS. Cells were permeabilized in 0.2% Triton X-100 (Solarbio) in PBS for 20 min at room temperature and washed three times with PBS. Then the cells were blocked for 30 min in 2% BSA (BioFroxx) in PBS. After the above treatment, cells were incubated in primary antibody (1:500, OTOP1 antibody, orb185690, Biorbyt; 1:200, PKD2L1 antibody, orb352708, Biorbyt) at 4 °C overnight. After extensive washing in PBS, Cells were mounted with Aqua-Poly/Mount (18606-20, Polysciences). Images were acquired on an Olympus Flouview 1000 confocal microscope. Related to Fig. 4a.

### Reproducibility

The number of experiments (patches) n for some figure panels is listed below. Figure 1c, *n*  =  5 for 1 × 10^−7^ M Zn^2+^, *n*  = 5 for 1 × 10^−6^ M Zn^2+^; *n*  =  3 for 1 × 10^−5^ M Zn^2+^; *n*  =  3 for 1 × 10^−4^ M Zn^2+^; *n*  =  5 for 1 × 10^−3^ M Zn^2+^. Figure 1e, *n*  =  9 for pH 8.00, *n*  =  7 for pH 8.25, and pH 8.50; *n*  =  5 for pH 8.75. Figure 1j, *n*  =  4 for pH 8.0, 9.5, 10.0, and 10.5; *n*  =  6 for pH 8.5; *n*  =  5 for pH 9.0. Figure 3e, untrans: *n*  =  4 for Na^+^, *n*  =  6 for K^+^, *n*  =  7 for Li^+^, *n*  =  4 for Cs^+^; mOTOP1: *n*  =  5 for Na^+^, *n*  =  3 for K^+^, *n*  =  3 for Li^+^, *n*  =  6 for Cs^+^. The experiments in Figs. 1f and 4a were repeated at least three times independently with similar results.

### Data analysis, statistics, and generation of structural model

Multiple sequence alignment and maximum-likelihood phylogenetic analysis were performed using MEGA-X software^35^. The structural model of mOTOP1 was generated in AlphaFold v2.0^28^. All experiments were repeated at least three times independently. Off-line data analysis was performed using IgorPro v6.04. All statistical values are shown as means ± SEM, and the *n* value represents the sample size of the experiment. Statistical analysis was performed using GraphPad Prism 9 (GraphPad Software Inc). *p < 0.05, **p < 0.01, ***p < 0.001, ****p < 0.0001.

### Reporting summary

Further information on research design is available in the Nature Portfolio Reporting Summary linked to this article.

## Supplementary information

Supplementary Information

Reporting Summary

## Data Availability

The data that support this study are available from the corresponding authors upon reasonable request. All data are contained within the article and supplementary information files. The source data underlying Figs. 1b, c, e, f–h, j, 2i, j, k, 3e, f, 4a, and Supplementary Figs. 1b, 2f, 3b–d, 4a, b, 5e, 7b, c, 8 are provided as the Source Data Files. Source data are provided with this paper.

## Source data

Source Data
