# Supplementary material for: Vertebrate OTOP1 is also an alkali-activated channel
Source: Nat Commun. 2023 Jan 3;14:26. doi: 10.1038/s41467-022-35754-9 (PMC9810603; doi:10.1038/s41467-022-35754-9)
Supplement: Supplementary file 1 — Supplementary Information [file 41467_2022_35754_MOESM1_ESM.pdf]

Vertebrate OTOP1 is also an alkali-activated channel

Supplementary information

Lifeng Tian[1,2,3,4,5,8], Hao Zhang[1,2,3,8], Shilong Yang[6,8], Anna Luo[1,2,3], Peter Muiruri Kamau[1,2,3,7], Jingmei Hu[1,2,3], Lei Luo[1,2*] & Ren Lai[1,2,4,7*]

[1]Key Laboratory of Animal Models and Human Disease Mechanisms of the Chinese Academy of Sciences/Key Laboratory of Bioactive Peptides of Yunnan Province/National & Local Joint Engineering Center of Natural Bioactive Peptides, Kunming Institute of Zoology, Chinese Academy of Sciences, Kunming 650223, Yunnan, China

[2]National Resource Center for Non-Human Primates, Kunming Primate Research Center/National Research Facility for Phenotypic & Genetic Analysis of Model Animals (Primate Facility), Kunming Institute of Zoology, Chinese Academy of Sciences, Kunming 650107, Yunnan, China.

[3]University of Chinese Academy of Sciences, Beijing 100049, China.

[4]School of Molecular Medicine, Hangzhou Institute for Advanced Study, University of Chinese Academy of Sciences, Hangzhou, 310024, China

[5]The cancer Hospital of the University of Chinese Academy of Sciences, Institute of Basic Medicine and Cancer (IBMC), Chinese Academy of Sciences, Hangzhou 310022, China.

[6]College of Wildlife and Protected Area, Northeast Forestry University, Harbin 150040, China

[7]Sino-African Joint Research Center, Kunming Institute of Zoology, Chinese Academy of Sciences, Kunming, Yunnan 650223, China.

[8]These authors contributed equally: Lifeng Tian, Hao Zhang, Shilong Yang.

*These authors jointly supervised this work: Lei Luo (luolei@mail.kiz.ac.cn) and Ren Lai (rlai@mail.kiz.ac.cn)

This file includes: Supplementary Figures 1-8.

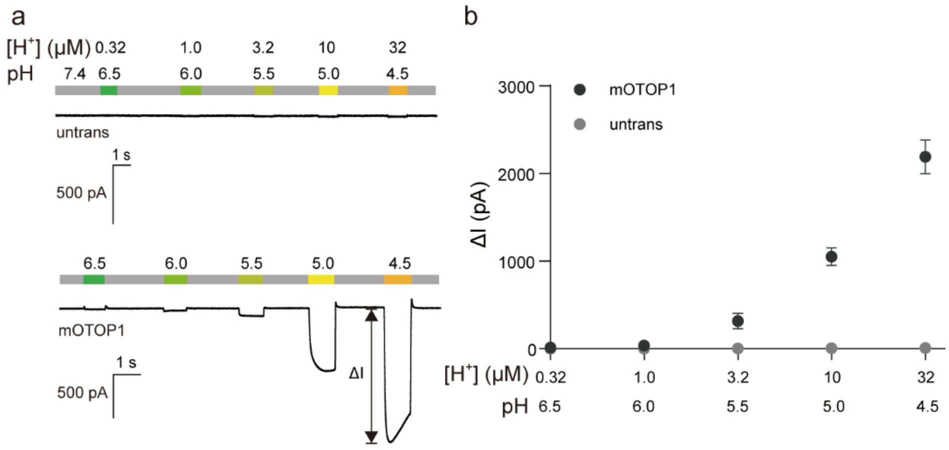

Supplementary Fig. 1: Acid activation of mOTOP1 channel. **a** Representative current traces evoked in HEK293T cells expressing mOTOP1 and untransfected HEK293T cells under different acidic pH OTOP1 extracellular solutions. **b** Plot of currents (ΔI; mean ± SEM) versus pH (6.5–4.5) in HEK293T cells expressing mOTOP1 channels (black dots, n = 11) and untransfected (gray dots, n = 10), with membrane potential held at 0 mV, mean ± SEM. Source data are provided as a Source Data file.

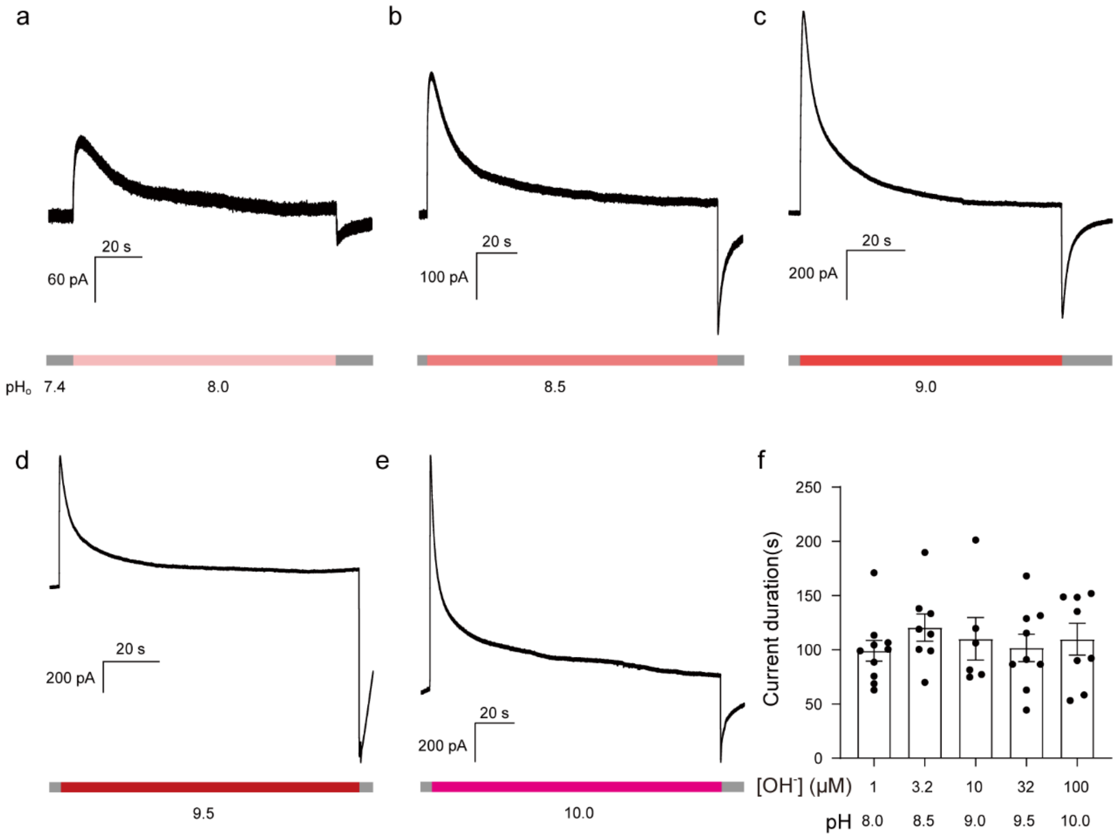

Supplementary Fig. 2: Current duration activated by alkali in HEK293T cells expressing mOTOP1 channels. **a-e** Representative alkali-induced currents with long-term alkali (pH 8.0-10.0) application at a holding potential of 0 mV. **f** Demonstration of the duration (from alkali-activated current generation to current holding constant) of outward current under long-term alkali (pH 8.0-10.0) application with different pH extracellular solutions (individual data point, mean ± SEM). pH 8.0: 99.055 ± 9.629 s, n = 10; pH 8.5: 120.468 ± 12.486 s, n = 8; pH 9.0: 110.077 ± 19.623 s, n = 6; pH 9.5: 101.671± 12.685 s, n = 9; pH 10.0: 109.737 ± 14.635 s, n = 8. One-way ANOVA, Dunnett's multiple comparisons test. There is no significant difference compared to pH 8.0. pH 8.5 vs. pH 8.0, $p = 0.6961$; pH 9.0 vs. pH 8.0, $p = 0.9789$; pH 9.5 vs. pH 8.0, $p = 0.9998$; pH 10.0 vs. pH 8.0, $p = 0.9735$. Source data are provided as a Source Data file.

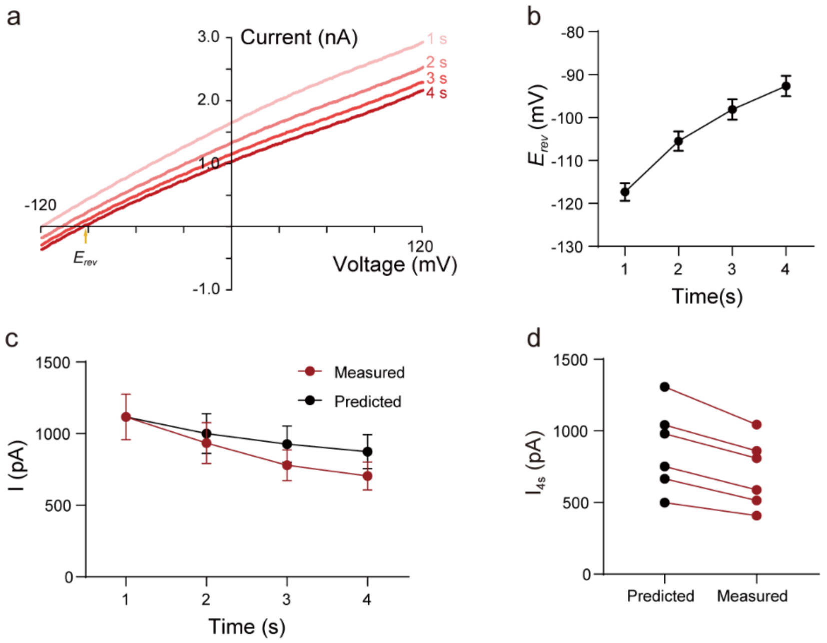

Supplementary Fig. 3: Changes in E_{rev} of mOTOP1 currents during long-term alkali application. **a** I-V curves of mOTOP1 channels were recorded at every one second interval in the alkaline extracellular solution. V_m was held at −120 mV and ramped to +120 mV, 1 V/s. **b** Change in E_{rev} as a function of time for OTOP1. Data are presented as mean ± SEM. 1 s: −117.323 ± 2.044 mV, n = 6; 2 s: −105.470 ± 2.250 mV, n = 6; 3 s: −98.095 ± 2.372 mV, n = 6, 4 s: −92.628 ± 2.402 mV, n = 6. **c-d** Comparison of the measured magnitude of currents at V_m = 0 mV predicted if the initial current decays due to a change in driving force for H^+ efflux. $I_{(x)} = I_{1s} *(V_m-E_{rev(x)})/(V_m-E_{rev(1s)})$, where x is a time point between 2 and 4 s after the onset of the stimulus. The measured current magnitudes were lower than the predicted calculated from the change in driving force. n = 6. Data are presented as mean ± SEM in (**c**). Source data are provided as a Source Data file.

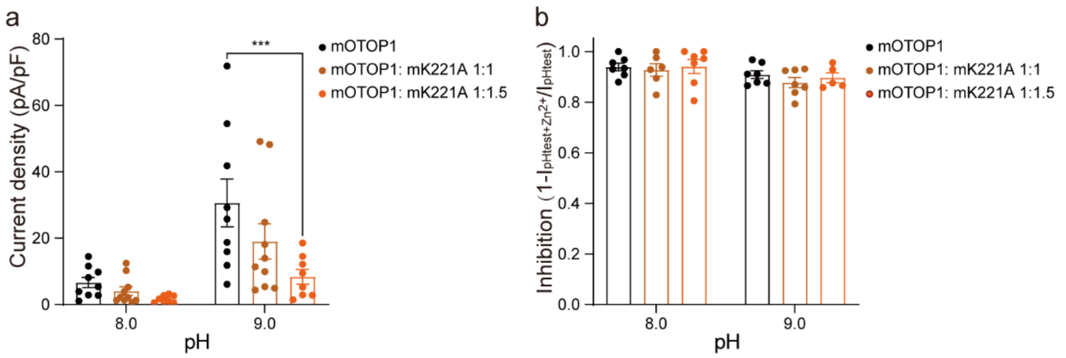

Supplementary Fig. 4: Alkali -induced current density of mOTOP1 channel correlates with its expression level. **a** Alkali -induced current density of mOTOP1 channel co-expressed with point mutants mK221A. Two-way ANOVA, Dunnett's multiple comparisons test, mean ± SEM. pH 8.0: mOTOP1 (n = 9) vs. mOTOP1:mK221A 1:1 (n = 10), p = 0.8530, mOTOP1 vs. mOTOP1:mK221A 1:1.5 (n = 8), p = 0.5996; pH 9.0: mOTOP1 (n = 9) vs. mOTOP1:mK221A 1:1 (n = 10), p = 0.0728, mOTOP1 vs. mOTOP1:mK221A 1:1.5 (n = 8), p = 0.0008. **b** Inhibition efficiency of 1 mM Zn^{2+} on alkali-induced currents in mOTOP1 channels co-expressed with the point mutant mK221A. Two-way ANOVA, Dunnett's multiple comparisons test, mean ± SEM. pH 8.0: n = 7 for mOTOP1 and mOTOP1:mK221A 1:1.5, n = 6 for mOTOP1:mK221A 1:1; pH 9.0: n = 7 for mOTOP1 and mOTOP1:mK221A 1:1, n = 5 for mOTOP1:mK221A 1:1.5. There is no significant difference. Source data are provided as a Source Data file.

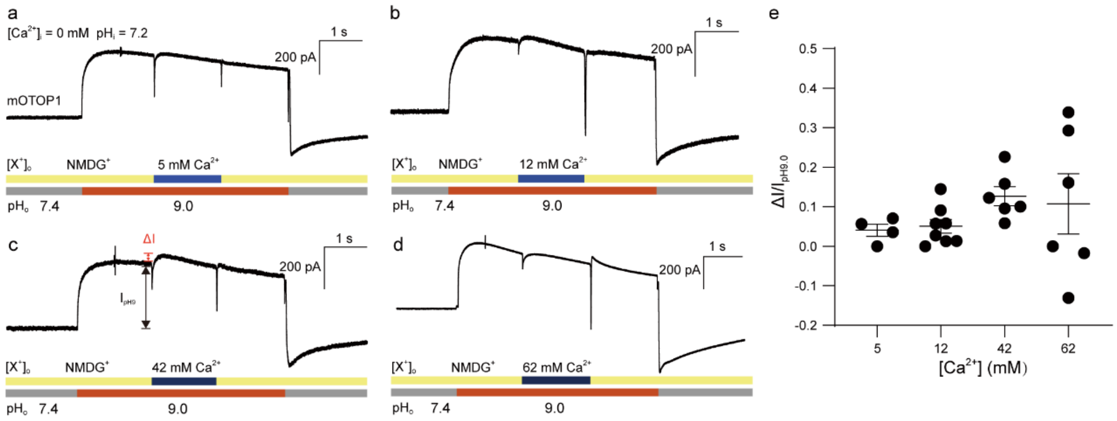

Supplementary Fig. 5: mOTOP1 channels are virtually impermeable to calcium ions when activated by alkali. **a-d** Calcium ions were chelated intracellularly with EGTA, $NMDG^+$ in the extracellular solution was replaced with gradient concentrations of Ca^{2+} to measure the permeability of mOTOP1 channels to calcium ions. **e** HEK293T cells expressing mOTOP1 channels are barely permeable to calcium ions at different extracellular calcium ion concentrations. Data are presented as mean ± SEM. A: 5 mM Ca^{2+}, n = 4; B: 12mM Ca^{2+}, n = 8; C: 42 mM Ca^{2+}, n = 6; D: 62mM Ca^{2+}, n = 6. One-way ANOVA, Dunnett's multiple comparisons test. 12 mM Ca^{2+} vs. 5 mM Ca^{2+}: p = 0.9965, 42 mM Ca^{2+} vs. 5 mM Ca^{2+}: p = 0.4110, 62 mM Ca^{2+} vs. 5 mM Ca^{2+}: p = 0.5940. Source data are provided as a Source Data file.

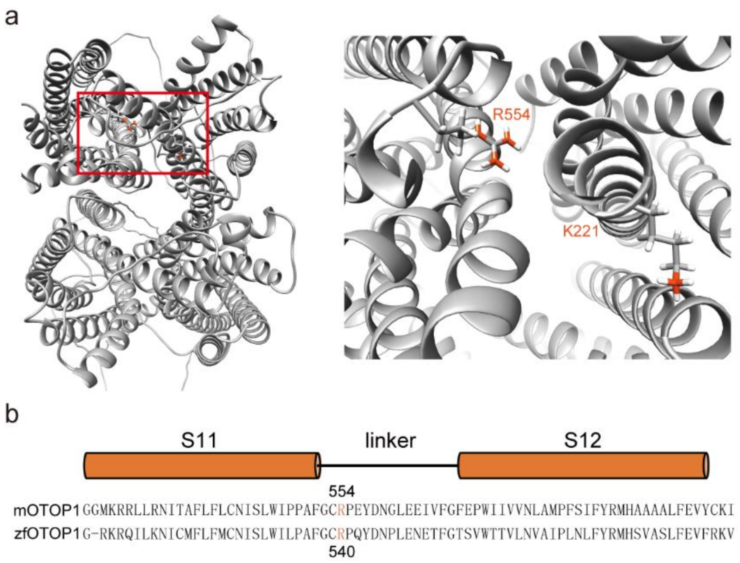

Supplementary Fig. 6: Key residues K221 and R554 in mOTOP1 are critical for alkali activation. **a** Top view of mOTOP1 structure (left), predicted by AlphaFold. Local view of K221 and R554, key residues of alkali-OTOP1 interactions (right). **b** Sequence alignment of mOTOP1 and zebrafish OTOP1 (zfOTOP1). Key residue associated with alkali binding is shown in orange. Cylinders above sequence indicate S11 and S12 transmembrane domains in OTOP1 structure.

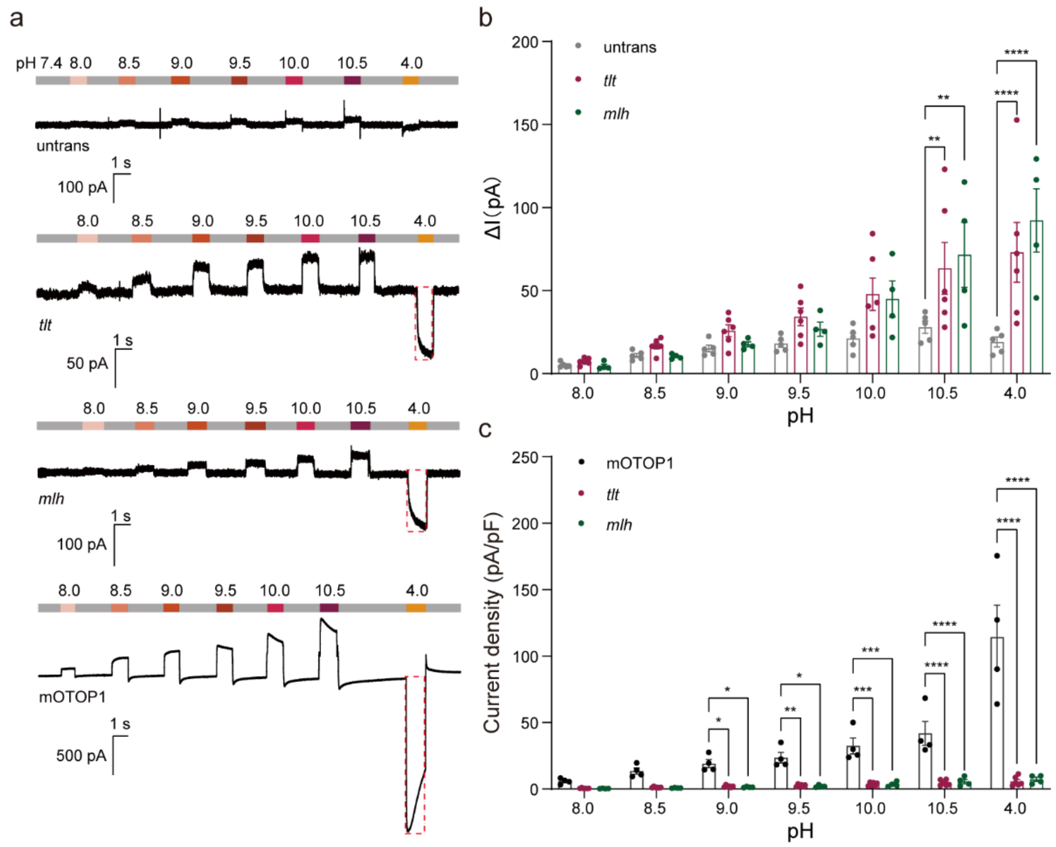

Supplementary Fig. 7: Alkali activation of naturally occurring mutations (tilted and mergulhador) of mOTOP1 channel. **a** Representative traces of currents evoked in HEK293T cells expressing mOTOP1, *tlt* (mA151E), *mlh* (mL408Q), and untransfected under different alkaline pH OTOP1 extracellular solutions. **b** Current amplitudes under different pH solutions in experiments of A, for *tlt* (n = 8), *mlh* (n = 4), and untrans (n = 5). Two-way ANOVA, Dunnett's multiple comparisons test, mean ± SEM. **c** Current magnitudes recorded under variable alkali concentrations were normalized to Capacitance to control for different cells, for *tlt* (n = 8), *mlh* (n = 4), and mOTOP1 (n = 4). Two-way ANOVA, Dunnett's multiple comparisons test, mean ± SEM. * $p < 0.05$, ** $p < 0.01$, *** $p < 0.001$, **** $p < 0.0001$. Exact p-values: **b**: ** $p = 0.0077$, ** $p = 0.0029$; **c**: * $p = 0.0298$, * $p = 0.0418$, ** $p = 0.0067$, * $p = 0.0117$, *** $p = 0.0002$, *** $p = 0.0006$. Source data are provided as a Source Data file.

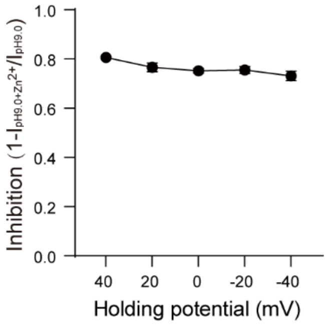

Supplementary Fig. 8: Inhibition efficiency of Zn^{2+} at different voltages. The inhibition efficiency of Zn^{2+} on mOTOP1 was not dramatically changed upon different stimulating voltages. One-way ANOVA, Dunnett's multiple comparisons test, n = 3 for 40 mV, 20 mV, and -40 mV, n = 4 for 0 mV and -20 mV. There is no significant difference compared to the inhibition rate at 0 mV. Source data are provided as a Source Data file.
