# Supplementary material for: Vertebrate OTOP1 is also an alkali-activated channel
Source: Nat Commun. 2023 Jan 3;14:26. doi: 10.1038/s41467-022-35754-9 (PMC9810603; doi:10.1038/s41467-022-35754-9)
Supplement: Supplementary file 2 — Reporting Summary [file 41467_2022_35754_MOESM2_ESM.pdf]

nature portfolio

Corresponding author(s): Ren Lai

Last updated by author(s): Dec 1, 2022

Reporting Summary

Nature Portfolio wishes to improve the reproducibility of the work that we publish. This form provides structure for consistency and transparency in reporting. For further information on Nature Portfolio policies, see our Editorial Policies and the Editorial Policy Checklist.

Statistics

For all statistical analyses, confirm that the following items are present in the figure legend, table legend, main text, or Methods section.

n/a	Confirmed	
☐	☒	The exact sample size (*n*) for each experimental group/condition, given as a discrete number and unit of measurement
☐	☒	A statement on whether measurements were taken from distinct samples or whether the same sample was measured repeatedly
☐	☒	The statistical test(s) used AND whether they are one- or two-sided<br>*Only common tests should be described solely by name; describe more complex techniques in the Methods section.*
☒	☐	A description of all covariates tested
☐	☒	A description of any assumptions or corrections, such as tests of normality and adjustment for multiple comparisons
☐	☒	A full description of the statistical parameters including central tendency (e.g. means) or other basic estimates (e.g. regression coefficient) AND variation (e.g. standard deviation) or associated estimates of uncertainty (e.g. confidence intervals)
☐	☒	For null hypothesis testing, the test statistic (e.g. *F*, *t*, *r*) with confidence intervals, effect sizes, degrees of freedom and *P* value noted<br>*Give P values as exact values whenever suitable.*
☒	☐	For Bayesian analysis, information on the choice of priors and Markov chain Monte Carlo settings
☒	☐	For hierarchical and complex designs, identification of the appropriate level for tests and full reporting of outcomes
☒	☐	Estimates of effect sizes (e.g. Cohen's *d*, Pearson's *r*), indicating how they were calculated

Our web collection on statistics for biologists contains articles on many of the points above.

Software and code

Policy information about availability of computer code

Data collection	PatchMaster v2x53, Olympus Flouview 1000,MetaFluor Software,AlphaFold v2.0
Data analysis	office Excel 2019, Igor Pro 6.04, MEGA x64, GraphPad Prism v9,Adobe Illustrator 2021

For manuscripts utilizing custom algorithms or software that are central to the research but not yet described in published literature, software must be made available to editors and reviewers. We strongly encourage code deposition in a community repository (e.g. GitHub). See the Nature Portfolio guidelines for submitting code & software for further information.

Data

Policy information about availability of data

All manuscripts must include a data availability statement. This statement should provide the following information, where applicable:

- Accession codes, unique identifiers, or web links for publicly available datasets
- A description of any restrictions on data availability
- For clinical datasets or third party data, please ensure that the statement adheres to our policy

All data are contained within the article and supplementary information files. The source data underlying Fig 1b, 1c, 1e, 1f-h, 1j, 2i 2j-k, 3e-f, 4a, and Supplementary Figs. 1b, 2f, 3b-d, 4a-b, 5e, 7b-c, 8 are provided as the Source Data Files.

Human research participants

Policy information about studies involving human research participants and Sex and Gender in Research.

Reporting on sex and gender	n/a
Population characteristics	n/a
Recruitment	n/a
Ethics oversight	n/a

Note that full information on the approval of the study protocol must also be provided in the manuscript.

Field-specific reporting

Please select the one below that is the best fit for your research. If you are not sure, read the appropriate sections before making your selection.

☒ Life sciences ☐ Behavioural & social sciences ☐ Ecological, evolutionary & environmental sciences

For a reference copy of the document with all sections, see nature.com/documents/nr-reporting-summary-flat.pdf

Life sciences study design

All studies must disclose on these points even when the disclosure is negative.

Sample size	Experiments were performed at least three independent times. Sample size in all experiments were estimated based in previous published experiments. In this study, the statistic analysis was obtained using student t test, Mann-Whitney test and One/Two-way ANOVA, and the values represents means plus minus standard error of the mean.<br>An evolutionarily conserved gene family encodes proton-selective ion channels. DOI:10.1126/science.aao3264 (2018).<br>Structural motifs for subtype-specific pH-sensitive gating of vertebrate otopetrin proton channels. DOI: 10.1101/2022.03.01.482452.
Data exclusions	No data were excluded.
Replication	Since experiments were performed at least three independent times, all experimental findings were reproducible.
Randomization	Randomly selected samples and organisms were allocated into experimental groups.
Blinding	Since results and phenotypes of mice were so obvious, the blinding was unnecessary.

Reporting for specific materials, systems and methods

We require information from authors about some types of materials, experimental systems and methods used in many studies. Here, indicate whether each material, system or method listed is relevant to your study. If you are not sure if a list item applies to your research, read the appropriate section before selecting a response.

Materials & experimental systems

n/a	Involved in the study	
☐	☒	Antibodies
☐	☒	Eukaryotic cell lines
☒	☐	Palaeontology and archaeology
☐	☒	Animals and other organisms
☒	☐	Clinical data
☒	☐	Dual use research of concern

Methods

n/a	Involved in the study	
☒	☐	ChIP-seq
☒	☐	Flow cytometry
☒	☐	MRI-based neuroimaging

Antibodies

Antibodies used	OTOP1 antibody, orb185690, Biorbyt,Tested Applications: ELISA, ICC, IF, IHC-P.<br>PKD2L1 antibody, orb352708, Biorbyt, Tested Applications: ELISA, IHC-P
Validation	The rabbit polyclonal antibody to OTOP1 (which react with human, mouse, rat species) was validated by the Biorbyt, and applied to

ELISA, ICC, IF, and IHC-P assays. The rabbit polyclonal antibody to PKD2L1 was validated by us in mouse PKD2L1-expressing HEK293T cells and untransfected cells by immunofluorescence assay.

Eukaryotic cell lines

Policy information about cell lines and Sex and Gender in Research

Cell line source(s)	HEK293T cells were purchased from Kunming Cell Bank, Kunming Institute of Zoology, Chinese Academy of Sciences (ATCC, CRL-3216).
Authentication	None of the cell line used were authenticated.
Mycoplasma contamination	Negative.
Commonly misidentified lines (See ICLAC register)	The study does not use misidentified lines.

Animals and other research organisms

Policy information about studies involving animals; ARRIVE guidelines recommended for reporting animal research, and Sex and Gender in Research

Laboratory animals	8–12 weeks C57BL/6L mice were used as wild type mice. Both sexes were used in this study. A 12-hour light/12-hour dark cycle is used, ambient temperatures is~18-23°C with 40-60% humidity.
Wild animals	Wild animals were not involved in this study.
Reporting on sex	Our findings apply to both gender. Both male and female mice were used.
Field-collected samples	No samples were collected from field.
Ethics oversight	All the animal experiments were carried out in strict accordance with recommendations in the Guide for the Care and Use of Laboratory Animals of Kunming Institute of Zoology, Chinese Academy of Sciences. Protocols were approved by the Institutional Animal Care and Use Committees at Kunming Institute of Zoology, Chinese Academy of Sciences (IACUC No.: IACUC-RE-2022-08-015). All possible efforts were employed to reduce the number of animals used and also to minimize animal suffering.

Note that full information on the approval of the study protocol must also be provided in the manuscript.
